# HMGB2 drives tumor progression and shapes the immunosuppressive microenvironment in hepatocellular carcinoma: insights from multi-omics analysis

**DOI:** 10.3389/fimmu.2024.1415435

**Published:** 2024-08-23

**Authors:** Yan-zhu Chen, Zhi-shang Meng, Zuo-lin Xiang

**Affiliations:** ^1^ Department of Radiation Oncology, Shanghai East Hospital, School of Medicine, Tongji University, Shanghai, China; ^2^ Department of Ophthalmology, The Second Xiangya Hospital, Central South University, Changsha, China; ^3^ Department of Radiation Oncology, Shanghai East Hospital Ji’an hospital, Ji’an, China

**Keywords:** hepatocellular carcinoma, HMGB2, multi-omics approaches, computational biology and bioinformatics, tumor microenvironment, immunosuppression

## Abstract

**Background:**

Hepatocellular carcinoma (HCC) poses a significant health burden globally, with high mortality rates despite various treatment options. Immunotherapy, particularly immune-checkpoint inhibitors (ICIs), has shown promise, but resistance and metastasis remain major challenges. Understanding the intricacies of the tumor microenvironment (TME) is imperative for optimizing HCC management strategies and enhancing patient prognosis.

**Methods:**

This study employed a comprehensive approach integrating multi-omics approaches, including single-cell RNA sequencing (scRNA-seq), bulk RNA sequencing (Bulk RNA-seq), and validation in clinical samples using spatial transcriptomics (ST) and multiplex immunohistochemistry (mIHC). The analysis aimed to identify key factors influencing the immunosuppressive microenvironment associated with HCC metastasis and immunotherapy resistance.

**Results:**

HMGB2 is significantly upregulated in HCC^Trans^, a transitional subgroup associated with aggressive metastasis. Furthermore, HMGB2 expression positively correlates with an immunosuppressive microenvironment, particularly evident in exhausted T cells. Notably, HMGB2 expression correlated positively with immunosuppressive markers and poor prognosis in HCC patients across multiple cohorts. ST combined with mIHC validated the spatial expression patterns of HMGB2 within the TME, providing additional evidence of its role in HCC progression and immune evasion.

**Conclusion:**

HMGB2 emerges as a critical player of HCC progression, metastasis, and immunosuppression. Its elevated expression correlates with aggressive tumor behavior and poor patient outcomes, suggesting its potential as both a therapeutic target and a prognostic indicator in HCC management.

## 1 Introduction

Hepatocellular carcinoma (HCC) stands as one of the most prevalent malignancies globally and represents a significant contributor to cancer-related mortality rates worldwide (1). The advent of immune checkpoint inhibitor (ICI)-based therapies has revolutionized the treatment landscape for HCC, offering promising prospects for patients across disease stages (2–4). Despite advancements in surgery, chemoradiotherapy, and immunotherapy, the management of HCC remains challenging due to its propensity for recurrence, therapy resistance, and metastasis (5, 6). Therefore, there is an urgent need to unravel the underlying mechanisms driving HCC progression and therapeutic resistance, in order to develop more effective treatment strategies and improve patient outcomes.

Characterized by considerable heterogeneity, the tumor microenvironment (TME) plays a pivotal role in HCC progression (7, 8). Comprising diverse cellular constituents including immune cells, stromal cells, and tumor cells, the TME orchestrates complex interactions that profoundly influence tumor growth, immune evasion, and therapy resistance (9, 10). Within this milieu, interactions between T cells and various cell types give rise to dynamic immunosupportive or immunosuppressive conditions, profoundly influencing tumor progression (11). In the context of cancer and chronic infection, CD8+ T cells may undergo exhaustion or dysfunction, marked by the upregulation of immune checkpoints like PD-1(PDCD1), CTLA4, LAG3, TIGIT, and TIM3(HAVCR2), impairing their anti-tumoral function (12, 13). This state of T cell exhaustion presents a significant challenge in cancer immunotherapy, as it is associated with reduced efficacy of ICIs and adoptive T cell therapies (2). Therefore, understanding the intricacies of the TME is imperative for tailoring targeted therapies to individual patients, thereby improving prognosis and treatment outcomes in HCC management.

The advent of single-cell RNA sequencing (scRNA-seq) analysis has revolutionized the understanding of the TME. With the ability to dissect cellular heterogeneity at single cell resolution, scRNA-seq has become a cornerstone in cancer research (14). The comprehensive mapping of TME components in HCC is continuously evolving, encompassing T cells (15), neutrophils (16), NK cells (17), as well as various potentially significant subsets and driver genes previously undiscovered, such as the function and clinical relevance of exhausted CD8+ T cells and SPP1+ tumor-associated macrophages within the TME (13). Moreover, the spatial transcriptomics (ST) has further enriched the comprehension of the TME by providing crucial spatial context to molecular analyses (18). For instance, recent findings have unveiled the spatial organization of the tumor immune barrier in HCC, which obstructs immunotherapy efficacy by hindering immune cell infiltration into malignant regions, highlighting the intricate interplay between spatial architecture and immunotherapeutic responses in the TME (19). These cutting-edge technologies empower researchers to investigate the TME with unprecedented depth and precision, leveraging algorithm-driven computational analyses and data-driven insights, these methodologies facilitate the exploration of tumor heterogeneity and the elucidation of intricate molecular networks underlying tumorigenesis and therapeutic resistance.

High-mobility group box 2 (HMGB2) is a member of the high-mobility group box family of proteins, which are highly conserved nuclear proteins involved in various cellular processes, including DNA repair, transcriptional regulation, and chromatin remodeling (20). In HCC, HMGB2 has been associated with poor prognosis and aggressive tumor behavior by altered cell proliferation through antiapoptotic pathways (21). However, its role in the immunosuppressive microenvironment of HCC remains largely unexplored.

In this study, we utilized multi-omics approaches encompassing scRNA-seq, Bulk RNA-seq, ST, and validation in clinical sample using multiplex immunohistochemistry (mIHC). Our principal aim is to elucidate the pivotal factors contributing to the immunosuppressive microenvironment associated with HCC metastasis and resistance to immunotherapy. By doing so, our study endeavors to pave the way for personalized treatment strategies customized to individual patients, thereby optimizing treatment effectiveness and improving overall patient outcomes in HCC.

## 2 Materials and methods

### 2.1 Patient sample collection and ethical statement

HCC tissue samples were obtained from patients undergoing surgical resection at Shanghai East Hospital. Tumor specimens were preserved by Paraformaldehyde fixation and paraffin embedding for subsequent analysis.

This study adhered to ethical guidelines as stipulated in the Declaration of Helsinki. Approval (No. 202308) was obtained from the Ethics Committee of Shanghai East Hospital. Informed consent was secured from all participants for the use of their tissue samples in research. Patient confidentiality was strictly maintained through anonymization of data and restricted access protocols.

### 2.2 Data acquisition

scRNA-seq analysis was conducted using data obtained from the GSE149614 dataset (22) within the Gene Expression Omnibus (GEO) database. Bulk RNA-seq analysis encompassed diverse datasets, including the TCGA-LIHC cohort (23) from The Cancer Genome Atlas (TCGA) (https://portal.gdc.cancer.gov/projects/TCGA-LIHC) and the ICGC-LIRI cohort from the International Cancer Genome Consortium (ICGC) (https://dcc.icgc.org/projects/LIRI-JP). Additional datasets sourced from the GEO database, including GSE144269 (24), GSE109211 (25),GSE104580(https://www.ncbi.nlm.nih.gov/geo/query/acc.cgi?acc=GSE104580),GSE14520 (26), and GSE54236 (27), were also incorporated. Integration of single-cell and spatial transcriptome analysis was facilitated by data from the GSE224411 dataset (28) within the GEO database. Immunohistochemical verification of related protein expression was obtained from the Human Protein Atlas (HPA) database (https://www.proteinatlas.org/).

### 2.3 Processing of scRNA-seq data

#### 2.3.1 Data preprocessing

The scRNA-seq data from the GSE149614 dataset encompassed 21 samples obtained from 10 HCC patients, including 10 primary tumors (PT), 8 non-tumor livers (NTL), 2 portal vein tumor thrombus (PVTT), and 1 metastatic lymph node (MLN). Initial data processing was conducted using the R package “Seurat” (29). Quality control were rigorously applied to exclude cells with gene expression levels outside the range of 500 to 8000, as well as those exhibiting mitochondrial gene expression exceeding 15%. Subsequently, gene expression values underwent normalization using the SCTransform method, followed by dimensionality reduction via principal component analysis (PCA). Inter-sample batch effects were corrected using the “Harmony” package (30). Clustering analysis was performed utilizing the FindNeighbors and FindClusters functions in Seurat. The results were visualized using Uniform Manifold Approximation and Projection (UMAP). Subclusters annotation was conducted using a combination of manual annotation based on recognized markers and automatic annotation using the “singleR” (31) and “sctype” (32) packages to ensure unbiased cell annotation. This process of dimensionality reduction, clustering, and subclusters annotation was consistently applied across epithelial/tumor cells, and NK/T cell subclusters. Additionally, the “copykat” package facilitated the inference of cell copy number variations (33), specifically focusing on distinguishing between non-malignant and malignant epithelial cells.

#### 2.3.2 Pseudotime trajectory analysis

The monocle 2 analysis (34) was utilized to explore the pseudotime trajectories between malignant and non-malignant epithelial subclusters, as well as between different CD8+ T cell subclusters. The “plot_cell_trajectory” and “plot_genes_in_pseudotime” functions were employed to visualize cell state trajectories and pseudotime processes, enabling the analysis of evolutionary dynamics. Following the identification of key branches, the results were visualized using the “plot_genes_branched_heatmap” function.

To elucidate the dynamic status and internal relationships among different subclusters of HCC cells, other three trajectory inference methods were employed. The VECTOR method, employing “quantile polarization” based on principal-component values, inferred cellular developmental trajectories (35). This analysis utilized the first 50 PCs, with default settings for other parameters, and results were visualized using UMAP plots with vector arrows indicating trajectory development directions. Additionally, the Slingshot method (36), based on dimensionality reduction and Gaussian mixture modeling generated by PCA, was utilized to infer evolutionary trajectories between different states of cells. Furthermore, CytoTRACE was employed to quantify the stemness and differentiation potential of computational cell subpopulations (37), facilitating the assessment of cellular states in terms of their relative stemness, thereby providing additional insights into the cellular hierarchy and developmental trajectories.

#### 2.3.3 Gene enrichment analysis

In the analysis of HCC cell subclusters, two complementary approaches were employed for gene enrichment analysis. Gene Ontology (GO) enrichment analysis was performed based on differentially expressed genes (DEGs) between subclusters using the “scRNAtoolVis” package (https://github.com/junjunlab/scRNAtoolVis). Including visualization of volcano plots of DEGs and heatmaps of marker genes and the top five most significant GO enrichment entries among HCC subpopulations were identified. Additionally, differences in cellular metabolic processes between subpopulations were analyzed and visualized using the scMetabolism package (38).

For the functional analysis of NK/T cells, the “AddModuleScore” function in Seurat was utilized. Specifically, acknowledged gene sets were used to compute cytotoxic scores for each cell (14, 39), including IFNG, GZMA, GZMB, GZMH, GZMK, GZMM, GNLY, PRF1, KLRC1, KLRB1, NKG7, KLRK1, KLRD1, FASLG, TNF, IL2, and LAMP1. Exhaustion scores were similarly computed using genes including PDCD1, CXCL13, TIGIT, CTLA4, HAVCR2, ICOS, CD274, LAYN, ENTPD1, BATF, TNFRSF9, TOX, LAG3, and ITGAE (40, 41). These scores were then integrated into visualizations in UMAP diagrams and violin plots.

#### 2.3.4 Cell communication analysis

To elucidate the cellular interactions between the HCC cells and T cells, we utilized the “CellChat” R package, which infers intercellular communication networks based on ligand-receptor interactions (42). Normalized gene expression data were used to identify potential ligand-receptor pairs across different cell types. The “CellChat” algorithm was employed to infer communication probabilities between cell types, identifying significant ligand-receptor interactions. Communication networks were visualized using circle plots, bubble plots, hierarchy plots, or heatmaps to depict the strength and frequency of interactions between HCC subpopulations and T cell subpopulations.

### 2.4 Processing of bulk RNA-seq data

All Bulk RNA-seq analyses were conducted using the BEST platform (43), a web application designed for comprehensive biomarker exploration in solid tumors on large-scale datasets.

#### 2.4.1 Prognostic analysis

The relationship between HMGB2 expression and HCC patient prognosis was investigated through Kaplan-Meier survival analysis. Patients were grouped based on the median expression level of HMGB2 in three cohorts: TCGA-LIHC, GSE144269, and ICGC-LIRI. Overall survival (OS), progression-free survival (PFS), disease-free survival (DFS) and disease-specific survival (DSS) were evaluated in the TCGA-LIHC cohort, while OS was assessed in the GSE144269 and ICGC-LIRI cohorts.

#### 2.4.2 Gene differential expression analysis

Differences in HMGB2 expression among patient samples of varying grades were compared in TCGA-LIHC using the Kruskal-Wallis test. HMGB2 expression was also compared between the responsive and non-responsive groups to sorafenib treatment in GSE109211, and between the transarterial chemoembolization (TACE) treatment responding and non-responding groups in GSE104580. Additionally, HMGB2 expression in tumor tissues versus normal tissues was examined in GSE144269, GSE14520, GSE54236, and TCGA-LIHC cohorts. Pairwise comparisons were conducted using the Wilcoxon test, with statistical significance set at *p* < 0.05.

#### 2.4.3 Gene set enrichment analysis

Gene set enrichment analysis based on GO terms was conducted using the “Enrichment analysis” function of the BEST platform. Initially, this tool calculated the correlation between the HMGB2 gene and all other genes across all HCC cohorts available on the platform. Subsequently, the average correlation coefficient across all cohorts was computed and the correlation coefficients were sorted in descending order. These correlation coefficients were then inputted into the Gene Set Enrichment Analysis (GSEA) algorithm (44), enabling unbiased enrichment analysis and providing valuable insights into the functional significance of HMGB2 across HCC cohorts.

#### 2.4.4 Correlation analysis of HMGB2 and immunosuppression gene expression

Correlation analysis was conducted to explore the relationship between HMGB2 and six immunosuppression-related genes, including PDCD1, CTLA4, HAVCR2, TIGIT, LAG3, and CD274 (PD-L1). The analysis was performed in both the TCGA-LIHC and ICGC-LIRI cohorts. Expression levels of all genes were normalized using the z-score method, Pearson correlation analysis was employed. Statistical significance was determined using a threshold of *p* < 0.05.

### 2.5 Processing of spatial transcriptome data

The investigation into the TME of HCC relies on ST analysis utilizing the GSE224411 dataset, which integrates both single-cell and ST data. Initially, scRNA-seq data underwent quality control, dimensionality reduction, clustering, and subcluster annotation, we then discern epithelial cells and NK/T cells based on HMGB2 expression status. Subsequently, we integrate these stratified subclusters with ST data using the FindTransferAnchors and TransferData functions. To unravel the complex spatial dynamics, we employ the “SPOTlight” package (45) for deconvolution analysis, enabling the visualization of each subcluster’s spatial distribution across the transcriptome landscape.

### 2.6 mIHC

Briefly, tissue slides underwent deparaffinization using xylene (2 times for 15 minutes each time), followed by rehydration through a graded series of ethanol dilutions (100%, 95%, 85%, and 75%, 5 minutes each). Antigen retrieval was performed using a microwave-based method with antigen restoration solution (10 mM citrate buffer, pH 6.0). To block endogenous peroxidase activity, slides were treated with 3% H_2_O_2_ for 15 minutes. Subsequently, nonspecific binding was minimized by incubating the sections in 10% normal goat serum (ZSGB-Bio, ZLI-9022) for 30 minutes. Primary antibodies against HMGB2 (1:200, Abclonal, A9168), GPC3 (1:100, Abclonal, A12383), CD8 (1:200, Abcam, ab237709), and PD-1 (1:200, Proteintech, 66220-1-Ig) were applied and incubated overnight at 4°C. Following primary antibody incubation, sections were incubated with the corresponding secondary antibodies. Signal amplification was achieved using a 4-color IHC kit according to the manufacturer’s instructions (AiFang Biological, AFIHC025). DAPI staining was employed to counterstain nuclei for 20 minutes. Finally, the stained slides were scanned using a digital scanner (3DHISTECH-Pannoramic MIDI, 3DHISTECH Ltd, Budapest).

## 3 Results

### 3.1 Summary of study design

**Figure 1:**
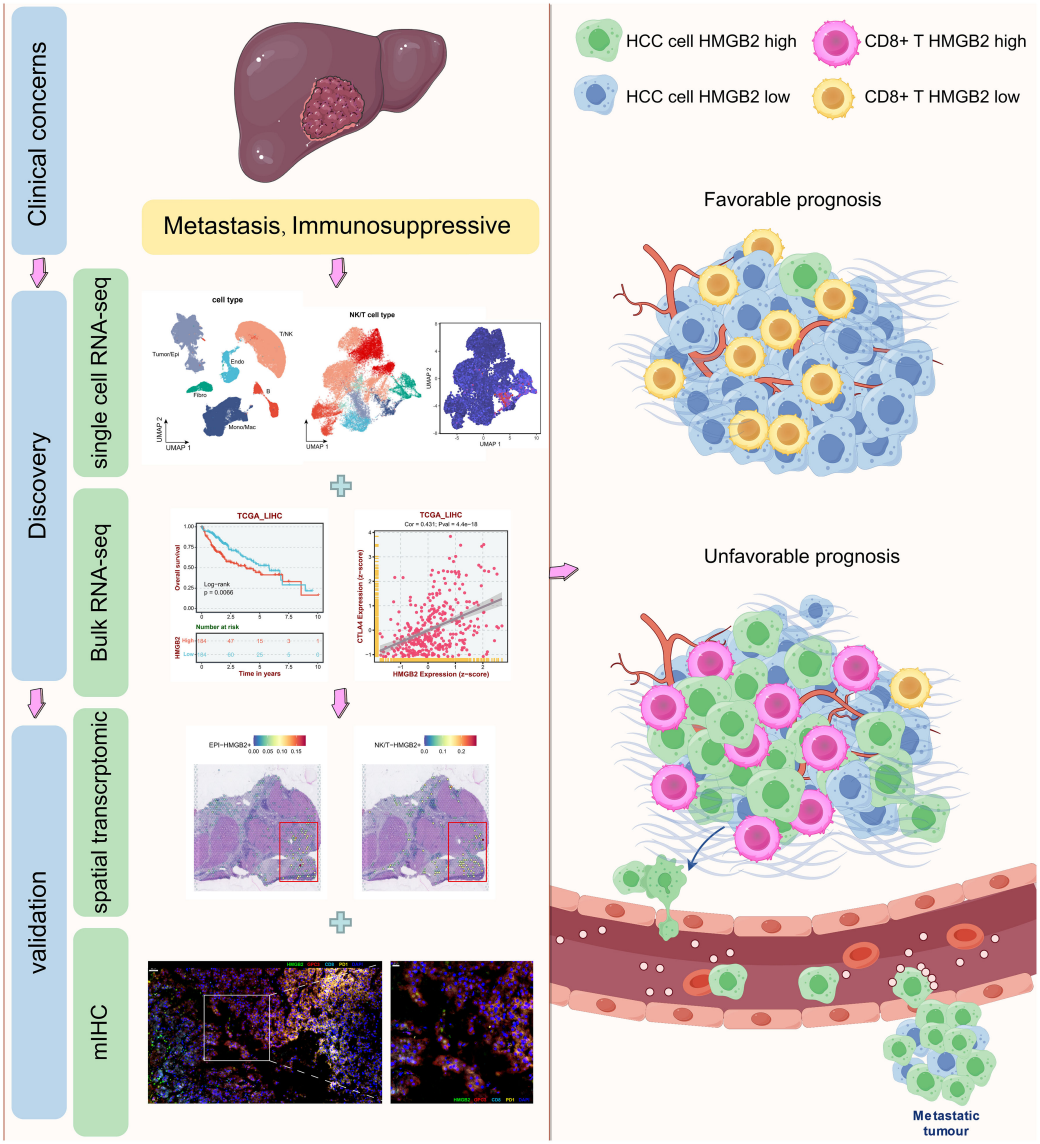
Graphical abstract of this study. Drawn and adapted by Figdraw (https://www.figdraw.com) and Smart Servier Medical Art (https://smart.servier.com/).

As shown in **Figure 1**, through an integrated approach encompassing multi-omics analyses, we discovered and validated the pivotal role of HMGB2 in tumor metastasis and the immunosuppressive microenvironment of HCC, highlighting its potential as a therapeutic target and prognostic marker for HCC management.

### 3.2 Identification of HMGB2 as a critical factor in hepatocyte transformation to HCC

**Figure 2:**
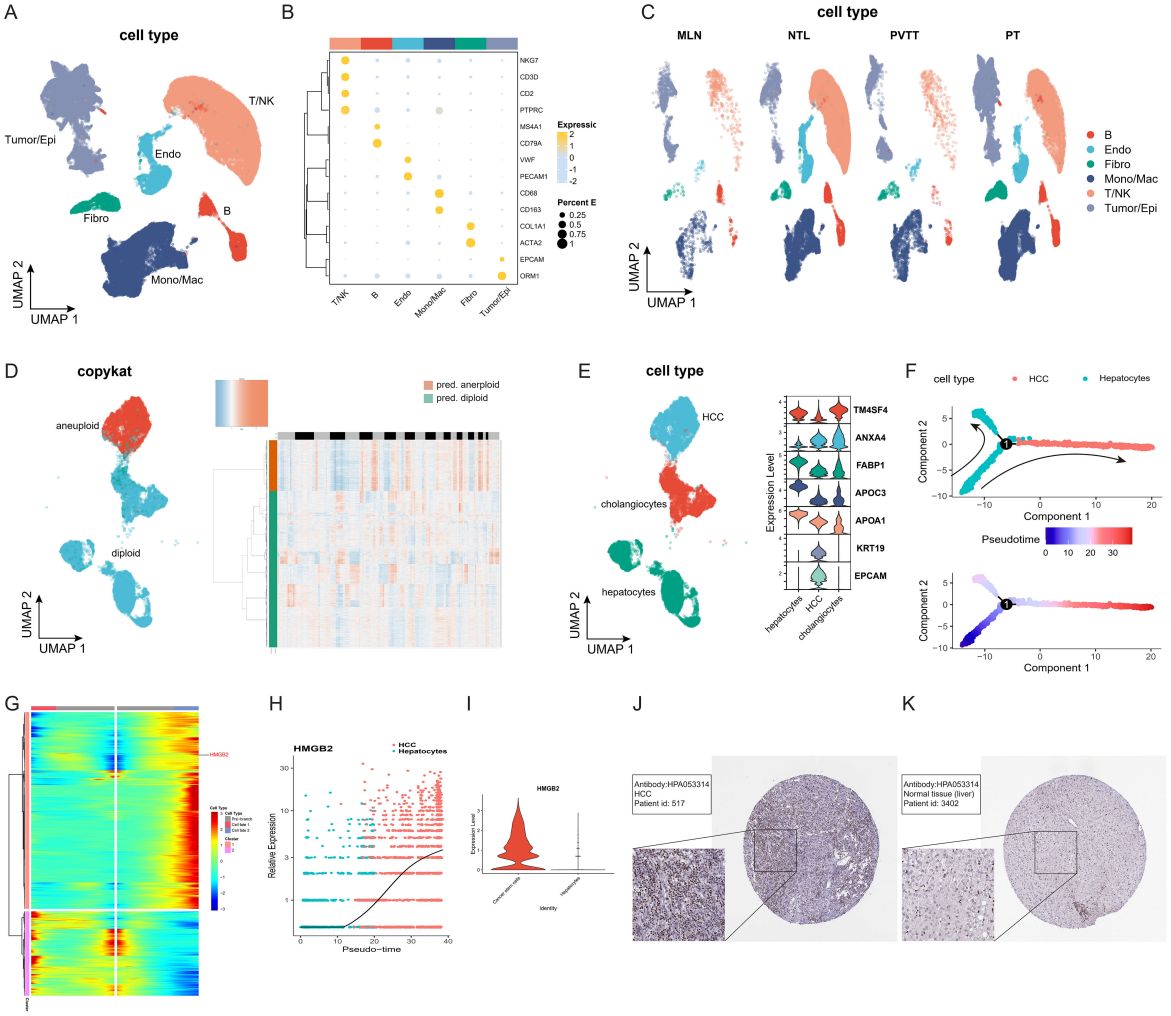
HMGB2 is a crucial factor in hepatocyte transformation to HCC cells. **(A)** UMAP diagram illustrating the distribution of subpopulations of all included cells. **(B)** Bubble chart depicting marker gene expression of each cell subpopulation. The darker the bubble color, the higher the expression level, and the larger the bubble, the greater the proportion of cells expressing the gene. **(C)** UMAP diagram showing the distribution of cell subpopulations of all cells among different clinical groups. **(D)** Left side: UMAP distribution map of cells with copykat judgment results. Right side: Heat map depicting copykat judgment results for each cell. **(E)** Left side: UMAP distribution map of three epithelial cell subpopulations (HCC, hepatocyte, cholangiocyte). Right side: Violin plot showing marker gene expression for each cell subpopulation. **(F)** Pseudotime trajectory diagram of the transformation process from hepatocytes to HCC cells. **(G)** Clustered heatmap of gene expression patterns across different cell fates after branch point 1. **(H)** Pseudotime expression of HMGB2 during hepatocyte transformation to HCC. **(I)** Violin plot illustrating the expression difference of HMGB2 between the hepatocyte group and the HCC group. **(J)** Immunohistochemical image of HMGB2 expression in HCC patients from the HPA database. **(K)** Immunohistochemical image of HMGB2 expression in normal liver tissue from the HPA database. Epi, epithelial cells; Endo, endothelial cells; Fibro, fibroblasts; NK, natural killer cells; Mono/Mac, monocytes/macrophages. PT, primary tumors; NTL, non-tumor livers; PVTT, portal vein tumor thrombus; MLN, metastatic lymph node.

Following quality control, batch removal, and standardization operations, a total of 68,097 cells meeting the criteria were included in subsequent analysis. The UMAP diagram depicted in **Figure 2A** vividly illustrates the distinct cell subpopulations, while the bubble chart in **Figure 2B** highlights differences in marker gene expression across these subpopulations. **Figure 2C** illustrates the distribution of subpopulations among the four clinical groups, providing further insights into their heterogeneity across clinical contexts. Subsequently, we conducted a comprehensive analysis of tumor/epithelial cell subpopulations, delving into their characteristics in greater depth. To delineate non-malignant and malignant epithelial cells, we combined two discernment methods: cells exhibiting CNV characteristics consistent with malignancy as determined by copykat-based assessment (**Figure 2D**) and those identified as HCC based on marker genes (**Figure 2E**) were classified as HCC cells. Subsequent pseudotime trajectory analysis of HCC cells delineated the progression process from hepatocytes to HCC cells (**Figure 2F**). Notably, analysis of branch point 1 revealed HMGB2 as an important influencing factor between different cell fates during malignant transformation of hepatocytes, evidenced by significantly differentially expressed genes (**Figure 2G**). Further examination indicated a substantial increase in HMGB2 expression in hepatocytes transitioning into HCC cells (**Figure 2H**), with significant differences observed in HMGB2 expression between hepatocytes and HCC cells (**Figure 2I**). Immunohistochemistry experiments based on the HPA database corroborated these findings, reaffirming HMGB2’s potential as a crucial factor in hepatocyte progression to HCC at the protein expression level (**Figures 2J, K**).

### 3.3 Identification of HMGB2 as a key contributor in HCC metastasis

**Figure 3:**
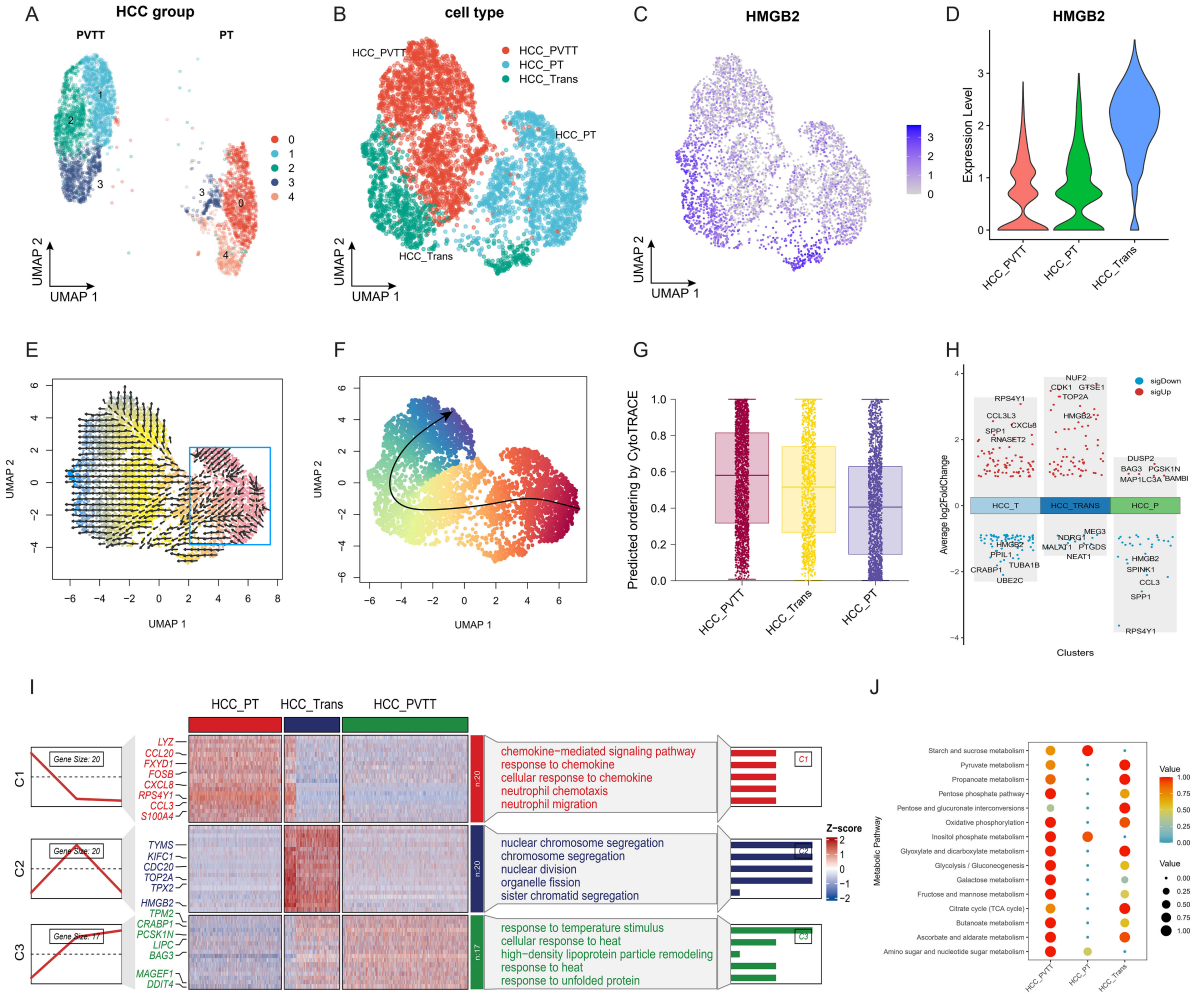
Role of HMGB2 in HCC Metastasis. **(A)** UMAP plot of HCC cells from PT and PVTT groups. **(B)** UMAP diagram illustrating the categorization of HCC cells into three distinct subgroups. **(C)** Feature plot highlighting elevated HMGB2 expression in HCC^Trans^ subgroups. **(D)** Violin plot demonstrating differential HMGB2 expression across the three HCC subgroups. **(E)** Trajectory inference on the UMAP plot reconstructed by VECTOR. **(F)** Trajectory inference on the UMAP plot using slingshot. **(G)** Boxplot illustrating the predicted ordering and CytoTRACE scores for the three HCC groups. **(H)** Volcano plot illustrating HMGB2 as a significant marker gene in HCC^Trans^. **(I)** Heatmap displaying differential gene expression patterns among the HCC subgroups, with different enrichment of GO biological processes among three subgroups. **(J)** Bubble chart demonstrating metabolic pathway distinctions among HCC subgroups.

We then proceeded to investigate the metastatic progression of HCC. HCC cells from groups of PVTT and PT were subjected to dimensional reduction and clustering, with each group exhibiting unique clusters. Notably, subcluster 3 was found to be shared between both groups, suggesting its pivotal role in the transformation process from primary tumor cells to those in the portal vein tumor thrombus (**Figure 3A**). Consequently, we categorized HCC into three subgroups: HCC^PVTT^, HCC^PT^, and HCC^Trans^ (Transition) (**Figure 3B**). Notably, HCC^Trans^ exhibited significantly higher HMGB2 expression compared to the other two groups (**Figures 3C, D**). Trajectory analyses using VECTOR (**Figure 3E**) and Slingshot (**Figure 3F**) demonstrated HCC^Trans^ as a critical transitional subgroup in the progression from HCC^PT^ to HCC^PVTT^. CytoTRACE-based quantification reaffirmed HCC^Trans^ as a crucial transitional state in HCC metastasis (**Figure 3G**). Differential gene expression analysis revealed HMGB2 as a significant marker gene in HCC^Trans^ (**Figure 3H**). Additionally, GO enrichment analysis highlighted distinct functional signatures, with HCC^PT^ enriched in immune responses, including responses to chemokines, neutrophil chemotaxis, and neutrophil migration, whereas HCC^Trans^ was predominantly associated with cell cycle and division processes, including chromosome segregation and nuclear division (**Figure 3I**). Metabolic profiling revealed metabolic pathway distinctions among the three HCC subgroups, with HCC^PT^ exhibiting the lowest metabolic pathway activity. Notably, HCC^Trans^ displayed the most active pyruvate metabolism, citrate cycle, and pentose and glucuronate interconversions, while HCC^PVTT^ demonstrated the highest activity in glycolysis, amino sugar, and nucleotide sugar metabolism (**Figure 3J**). These findings elucidate the pivotal role of HMGB2 in orchestrating HCC metastatic progression.

### 3.4 Identification of HMGB2 as a key factor in T cell exhaustion in HCC

**Figure 4:**
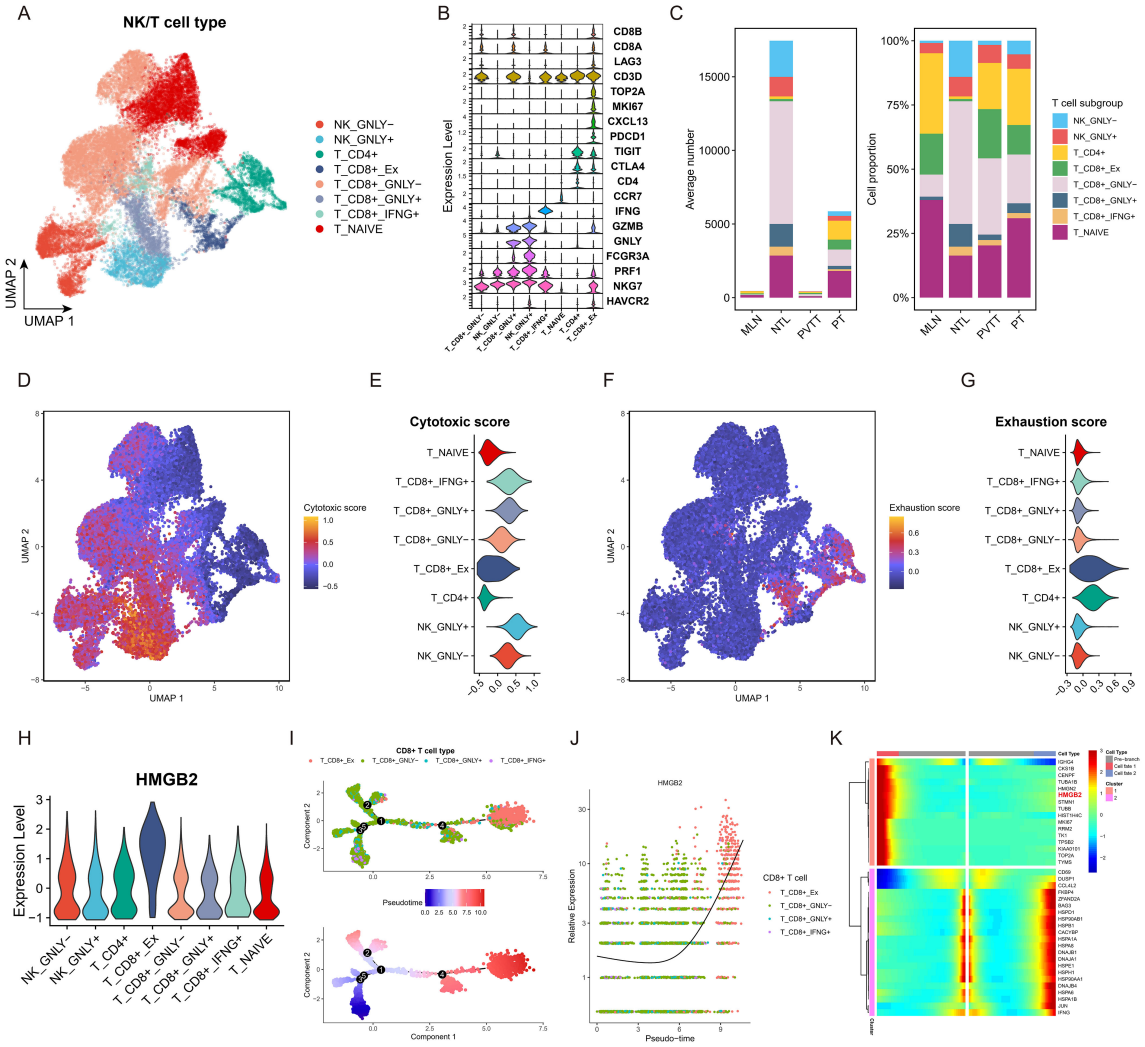
Role of HMGB2 in T cell exhaustion in HCC. **(A)** UMAP plot of NK/T cell subpopulations. **(B)** violin plot showing expression of recognized marker genes for each NK/T cell subpopulation. **(C)** Bar chart comparing the absolute numbers and relative proportions of each NK/T cell subset in the four clinical groups. **(D)** Feature plot of cytotoxic score on the UMAP diagram. **(E)** Violin plot displaying cytotoxic score in each NK/T cell subpopulation. **(F)** Feature plot of exhaustion score on the UMAP diagram. **(G)** Violin plot displaying cytotoxic score in each NK/T cell subpopulation. **(H)** Violin plot illustrating HMGB2 expression across NK/T cell subpopulations. **(I)** Pseudotime trajectory diagram of the transformation process of CD8+ T cells. **(J)** Pseudotime expression of HMGB2 during transformation process of CD8+ T cells. **(K)** Clustered heatmap of gene expression patterns across different cell fates after branch point 4.

We further characterized NK/T cell subpopulations through dimensionality reduction and clustering, as illustrated in the UMAP diagram in **Figure 4A**. Annotation into 8 distinct subpopulations was achieved based on established marker genes (**Figure 4B**). Quantitative analysis across clinical groups revealed a higher proportion of exhausted T cells in metastatic lesions compared to primary lesions in terms of both absolute numbers and proportions (**Figure 4C**). Further analysis demonstrated significantly higher cytotoxicity scores in IFNG+ CD8+ T, GNLY+ CD8+ T, and GNLY+ NK subpopulations (**Figures 4D, E**). Conversely, the exhausted T subgroup exhibited the highest exhaustion score (**Figures 4F, G**). Importantly, the exhausted T subgroup demonstrated the highest expression of HMGB2 among all subgroups. (**Figure 4H**). Pseudotime analysis of CD8+ T cells revealed the exhausted T subgroup to be situated at the terminal end of the developmental trajectory (**Figure 4I**), with HMGB2 expression increasing with pseudotime progression (**Figure 4J**). Analysis at key branch point 4 further underscored HMGB2 as a pivotal factor in the fate determination of CD8+ T cells transitioning into exhausted T phenotype (**Figure 4K**).

### 3.5 Cell-cell interaction in the HCC and T cells

**Figure 5:**
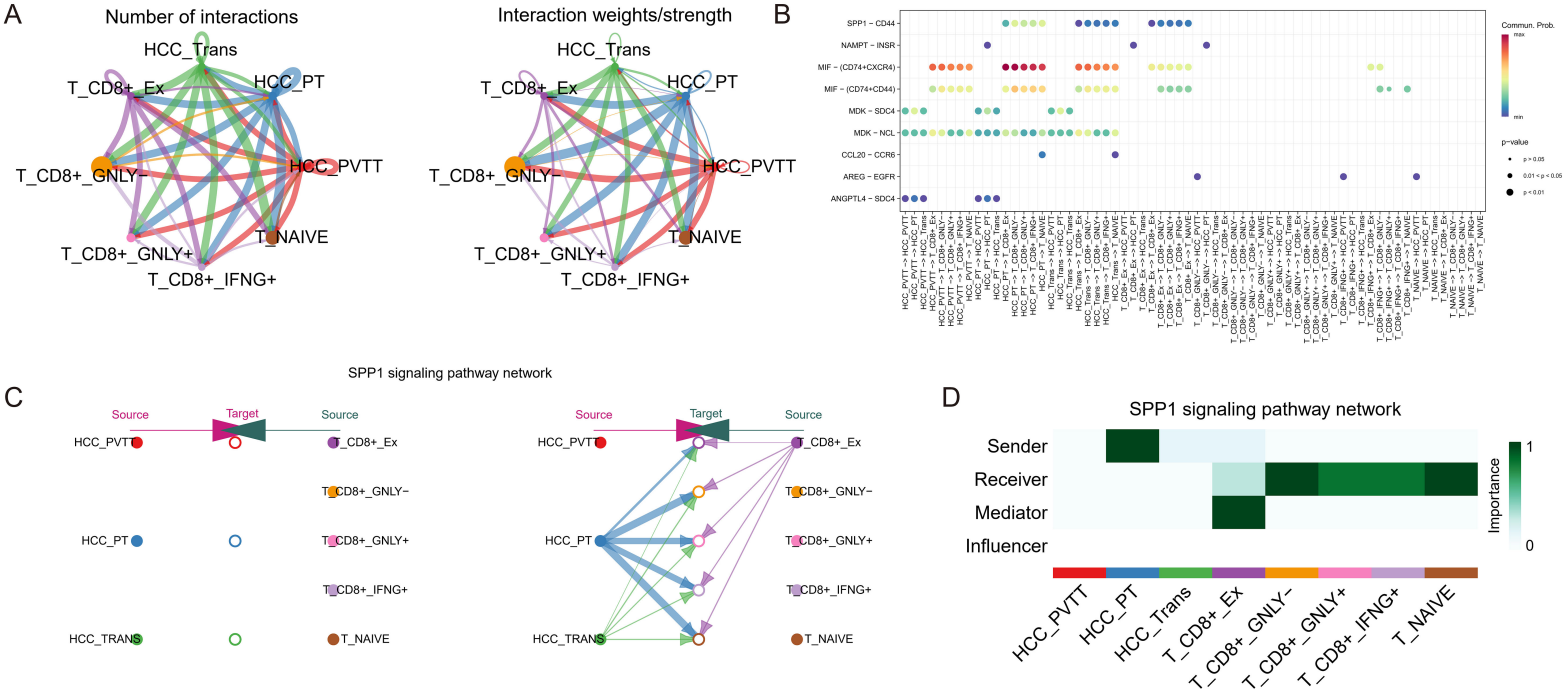
Cell-cell interaction analysis in HCC and T cells. **(A)** The number and strength of interaction pathways among eight cell types. **(B)** Bubble plot showing specific pathways of the eight cell-cell interactions. **(C)** Hierarchical plot showing the inferred intercellular communication network for SPP1 signaling, indicating the sources and targets among HCC and T cell subpopulations. **(D)** Heatmap depicting the SPP1 signaling pathways to evaluate each cell type’s involvement in cell interaction, showing the relative importance of each cell type as sender, receiver, mediator, and influencer based on the SPP1 signaling.

To elucidate the cellular interactions between HCC and T cell subpopulations, we performed cell communication analysis using CellChat. **Figure 5A** illustrates the number and strength of the cell-cell interaction pathways among the eight identified cell types. **Figure 5B** presents a bubble diagram of the specific signaling pathways involved in these interactions. The hierarchical diagram in **Figure 5C** demonstrates the SPP1 signaling pathway, with HCC^PT^, HCC^Trans^, and exhausted CD8+ T acting as primary sources. The SPP1-CD44 pathway interaction involves all T cell subpopulations as targets. The heatmap in **Figure 5D** shows that HCC^PT^ is the most potent sender, while exhausted CD8+ T is a prominent mediator, sender, and receiver, indicating complexinteractions that potentially contribute to an immunosuppressive microenvironment.

### 3.6 Investigating the prognostic implications of HMGB2 expression in HCC

**Figure 6:**
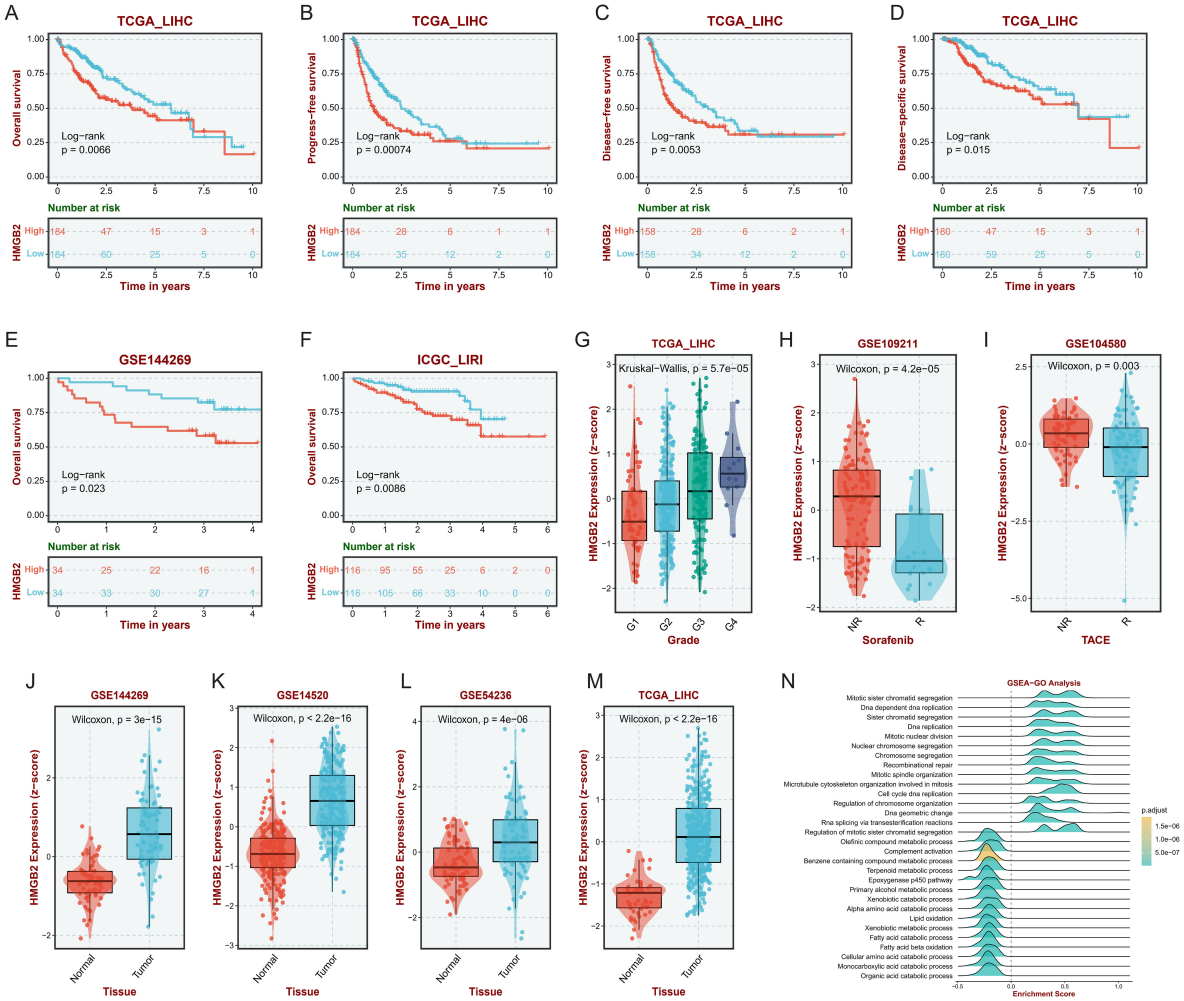
Impact of HMGB2 expression on prognosis and therapeutic response in HCC. Kaplan-Meier survival curves depicting OS **(A)**, PFS **(B)**, DFS **(C)** and DSS**(D)** in the TCGA-LIHC cohort stratified by HMGB2 expression levels. Kaplan-Meier survival curves illustrating OS in the GSE144269 **(E)** and ICGC-LIRI **(F)** cohorts based on HMGB2 expression levels. **(G)** Box plot demonstrating the association between HMGB2 expression and tumor grade in the TCGA-LIHC cohort. Comparative analysis of HMGB2 expression in responders vs. non-responders to sorafenib **(H)** and TACE **(I)** treatments in the TCGA-LIHC cohort. **(J–M)** Comparison of HMGB2 expression levels between tumor tissues and adjacent normal tissues in four independent cohorts. **(N)** Ridgeline plot illustrating GSEA results, highlighting significant positive correlations between HMGB2 expression and key biological processes.

Based on the HCC bulk RNA-seq data analysis in the BEST platform, it was observed that in the TCGA-LIHC cohort, the subgroup with high HMGB2 expression had poorer OS (**Figure 6A**), PFS (**Figure 6B**), DFS (**Figure 6C**), and DSS (**Figure 6D**) than the low-expression group. Similarly, OS in the high HMGB2 group was significantly worse in the GSE144269 cohort (**Figure 6E**) and ICGC_LIRI cohort (**Figure 6F**). Furthermore, in the TCGA-LIHC cohort, the expression of HMGB2 significantly increased with tumor grade, suggesting a potential association with tumor aggressiveness (**Figure 6G**). Additionally, HMGB2 expression was markedly higher in non-responders to sorafenib treatment compared to responders (**Figure 6H**), as well as in non-responders to TACE treatment (**Figure 6I**). Moreover, HMGB2 expression was consistently elevated in tumor tissues compared to normal tissues across multiple independent cohorts (**Figures 6J–M**). GSEA revealed a positive correlation between HMGB2 expression and critical biological processes such as cell cycle regulation, DNA replication, and sister chromatid segregation (**Figure 6N**), suggesting its potential involvement in tumor progression and prognosis.

### 3.7 Correlation analysis of HMGB2 and immunosuppressive genes in HCC

**Figure 7:**
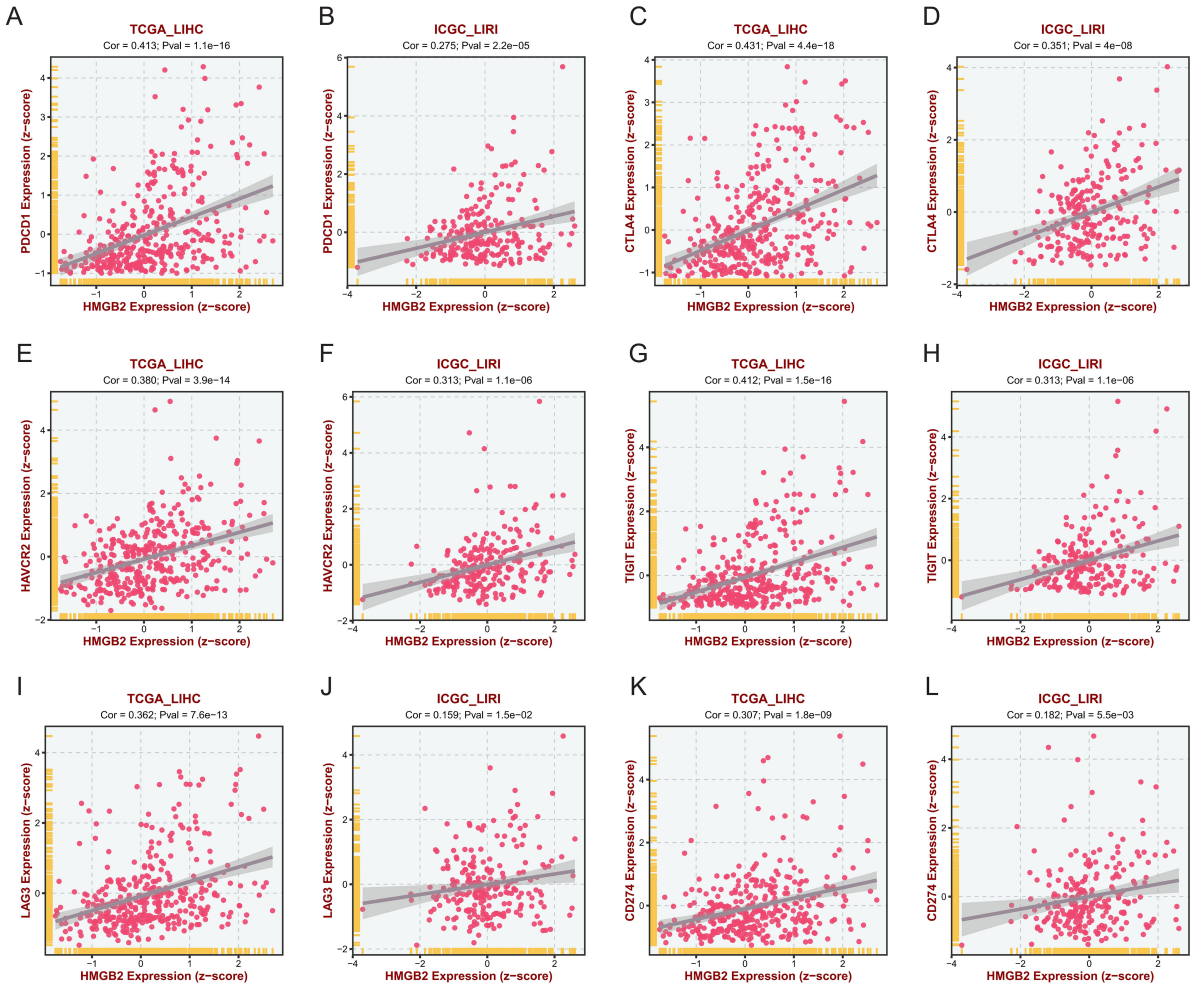
Correlation Analysis of HMGB2 and immunosuppressive genes in HCC. Scatter plots demonstrate the expression correlation between HMGB2 and various immunosuppressive genes in the TCGA-LIHC and ICGC-LIRI cohorts. Panels **(A–L)** present the correlation of HMGB2 expression with PDCD1, CTLA4, HAVCR2, TIGIT, LAG3, and CD274 in two cohorts respectively.

Correlation analysis was conducted to explore the relationship between HMGB2 expression and various immunosuppressive genes in HCC. Utilizing two bulk RNA-seq datasets, scatter plots were generated to depict the expression correlation between HMGB2 and six key immunosuppressive genes: PDCD1, CTLA4, HAVCR2, TIGIT, LAG3, and CD274. **Figures 7A–L** present the expression correlation scatter plots for each gene in both the TCGA-LIHC and ICGC-LIRI cohorts. Remarkably, all scatter plots demonstrated a significant positive correlation between HMGB2 expression and the expression of the immunosuppressive genes across both cohorts. These findings suggest a potential role for HMGB2 in contributing to the immunosuppressive microenvironment in HCC.

### 3.8 Role of HMGB2 in the immunosuppressive microenvironment of HCC

**Figure 8:**
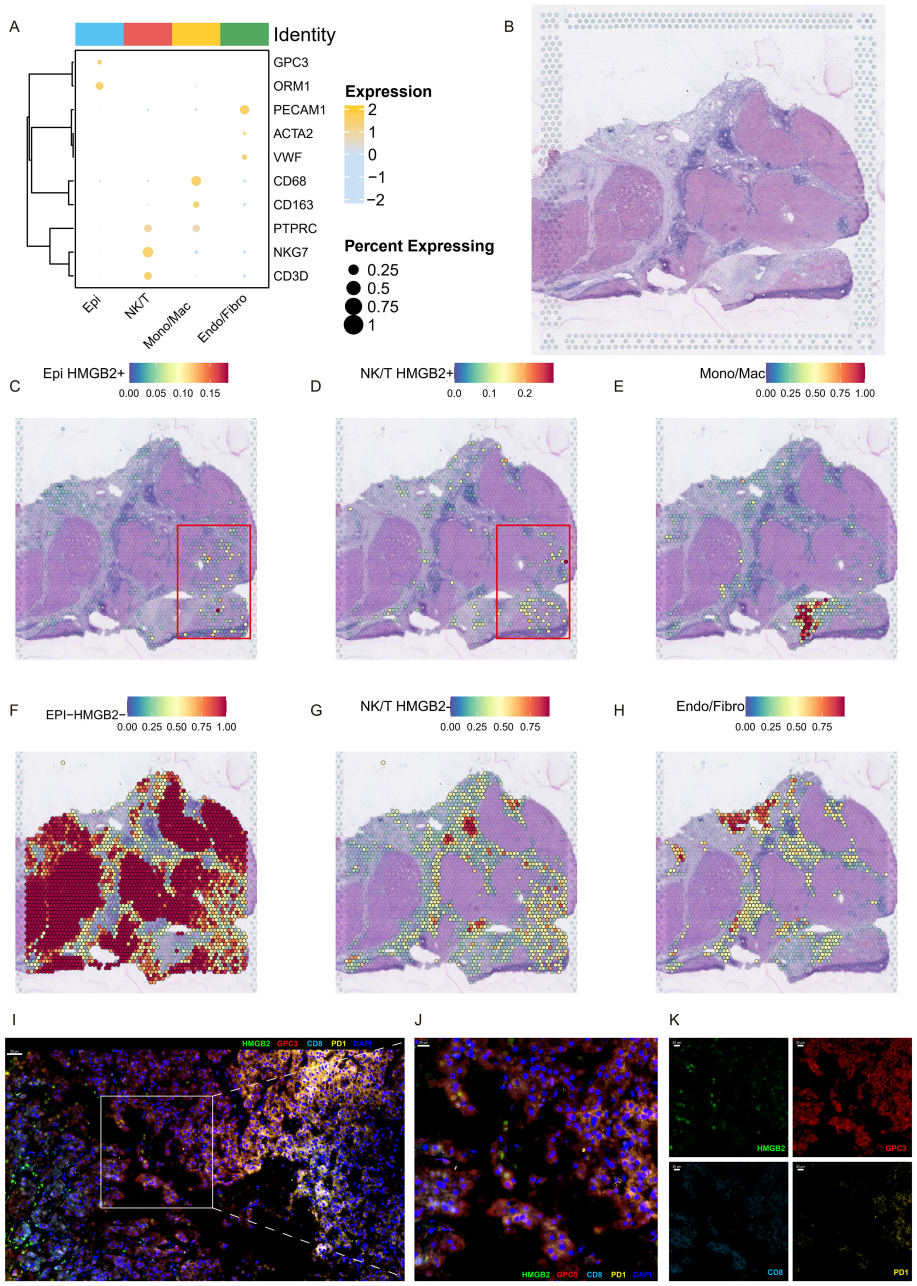
Spatial characterization of HMGB2 expression in the HCC microenvironment. **(A)** Bubble plot depicting the expression profiles of marker genes across subpopulations derived from scRNA-seq within the ST dataset. **(B)** H&E staining of ST section. Spatial deconvolution map delineating the localization patterns of **(C)** HMGB2+ epithelial cells, **(D)** HMGB2+ NK/T cells, **(E)** monocytes/macrophages, **(F)** HMGB2- epithelial cells, **(G)** HMGB2- NK/T cells, and **(H)** endothelial/fibroblast cells. **(I)** Representative image demonstrating the co-localization of HMGB2 (green), GPC3 (red), CD8 (cyan), and PD-1 (yellow) in TME of HCC tissues. **(J)** A partial enlargement of the image highlighting the co-expression of these markers. **(K)** Faceted fluorescence image displaying the four indicators: HMGB2, GPC3, CD8, and PD-1, further confirming their co-localization in the HCC microenvironment.

We then elucidate the intricate interplay between HMGB2 and the immunosuppressive microenvironment characterizing HCC leveraging ST analysis and mIHC techniques. **Figure 8A** presents a bubble plot illustrating the expression of recognized marker genes across cell subpopulations derived from scRNA-seq data in the ST dataset. **Figure 8B** depicts the ST atlas of the sample under study. ST analysis reveals the distribution of HMGB2+ epithelial cells, as depicted in **Figure 8C**. Additionally, **Figure 8D** showcases the spatial deconvolution map of HMGB2+ NK/T cells, demonstrating their spatial organization within the TME. Remarkably, HMGB2+ epithelial cells and HMGB2+ NK/T cells exhibit spatial co-localization. Further spatial mapping in **Figures 8E–H** delineates the distribution of monocytes/macrophages, HMGB2- epithelial cells, HMGB2- NK/T cells, and endothelial/fibroblast cells within the HCC microenvironment. Finally, the mIHC results, depicted in **Figures 8I–K**, provide additional insights into the spatial co-expression patterns of HMGB2, GPC3, CD8, and PD-1 within the TME, specifically, GPC3 staining highlighted the distribution of HCC cells, while CD8 and PD-1 staining co-localized with exhausted T cells. **Figure 8I** illustrates the comprehensive spatial distribution of these markers in the TME of HCC. As shown in **Figure 8J**, a partial enlargement of the image reveals the spatial co-expression of HMGB2 in specific HCC cells and T cell subsets. Notably, **Figure 8K** presents a faceted image showcasing the four indicators, providing detailed visualization of their expression patterns. Remarkably, the mIHC results underscore the presence of PD-1+ CD8 T cells infiltrating into HMGB2+ HCC cells, while concurrently expressing HMGB2 themselves. This reciprocal expression pattern elucidates the complex interplay between HCC cells and infiltrating T cells, shedding light on the dynamic nature of HMGB2-mediated immune modulation in HCC progression and immunosuppression.

## 4 Discussion

HCC exhibits significant heterogeneity, influencing its aggressiveness, metastatic propensity, and therapeutic responsiveness (46). Recent investigations have elucidated the divergence between AFP-negative and AFP-positive HCC subtypes, providing invaluable insights into the underlying mechanisms driving tumor progression (47). Additionally, studies conducted by Meng et al. have elucidated the pivotal role played by specific notably GPNMB+ Gal-3+ hepatic parenchymal cell subgroups, in driving hepatocellular carcinogenesis, thereby underscoring the complexity of the TME (48).

In this study, we adopted a stringent definition of malignant cells to achieve a more accurate understanding. Pseudotime analysis and differential expression profiling revealed HCC^Trans^, characterized by heightened HMGB2 expression, as pivotal in driving tumorigenesis and metastasis. Furthermore, our investigation uncovered the involvement of HMGB2 in CD8+ T cell exhaustion and the immunosuppressive microenvironment of HCC, highlighting its multifaceted role in hepatocarcinogenesis at single-cell resolution.

HMGB2 is expressed in all immortalized human and mouse cells, possibly helping to overcome replication limitations (49). Previous studies have linked HMGB2 to the development and progression of several cancers by influences cell proliferation, apoptosis, and metastasis through antiapoptotic pathways (21). Our study extends these findings by demonstrating that HMGB2 not only affects cell proliferation but also plays a significant role in the immunosuppressive microenvironment of HCC. The comprehensive analysis provided insights into the functional implications of HMGB2 through GO and metabolic pathway enrichment analyses, revealing its enrichment in pathways related to cell cycle/division regulation, pyruvate metabolism, and the citrate cycle, further corroborating the significance of HMGB2 in driving HCC progression. Targeting HMGB2 may disrupt these critical pathways involved in the metabolic reprogramming of cancer cells, thereby inhibiting tumor growth and progression, offering a promising avenue for improving patient outcomes.

Additionally, our study demonstrated that HMGB2 was associated with worse OS, DFS, and PFS in patients, HMGB2 expression was significantly higher in patients with advanced tumor progression or non-response to antitumor therapy. Notably, the evidence suggesting that blood levels of HMGB2 may serve as diagnostic markers for early-stage liver fibrosis and cirrhosis underscores its potential role in the early development of HCC (50), providing a window for preventive interventions aimed at mitigating disease progression. This emphasizes the dual role of HMGB2 as both a prognostic biomarker for HCC treatment and a candidate for early detection and prevention strategies.

Following the promising outcomes of the IMbrave150 trial, immunotherapy has emerged as a promising treatment modality for advanced HCC, although its efficacy remains limited to a subset of patients (3). The intricate interplay between HCC cells and the TME significantly influences disease progression and therapeutic outcomes. Recent data from diverse immunotherapy cohorts have underscored the significance of baseline tumor-intrinsic characteristics in determining the response to ICIs (51–53). Additionally, the presence of exhausted CD8+ T cells in TME, characterized by impaired effector function and proliferative capacity, has attracted considerable attention due to their involvement in immune evasion mechanisms, as reprogramming these cells could potentially enhance immunotherapy efficacy. Studies have identified HMGB2 as a crucial regulator of CD8+ T cell exhaustion during chronic viral infection and cancer, critical for their long-term maintenance (54). However, the role of HMGB2 in CD8+ T cell exhaustion in HCC and its relevance within the immunosuppressive microenvironment remain unexplored.

A recent spatial transcriptomic study involving HCC patients receiving anti-PD-1 therapy revealed a distinct tumor-immune barrier structure comprising SPP1+ macrophages and CAFs localized at tumor boundaries in non-responders (19), interestingly, HMGB2+ HCC cells were enriched around the tumor-immune barrier in non-responders (19), yet the role of HMGB2 in the immunotherapy resistance microenvironment of HCC was not investigated. Increasing evidence demonstrates that SPP1 pathway correlates with the local immunosuppressive microenvironment of tumors (55, 56). Our cell communication analysis found that the CD8+ exhausted T cell subset with high expression of HMGB2 plays an important role in the SPP1 pathway. This may serve as a crucial insight for future understanding of how HMGB2 promotes immunosuppression in the local immune microenvironment of HCC, thereby facilitating tumor invasion, metastasis, or resistance to immunotherapy. Moreover, we validated the spatial distribution and potential functional relevance of HMGB2 within the TME of HCC using ST analysis and mIHC. Our findings underscore the therapeutic potential of targeting HMGB2 in combination with immunotherapeutic approaches, offering promising avenues for synergistic HCC treatment strategies. Further investigations are warranted to explore the clinical utility of HMGB2-targeted therapies and their potential to enhance patient outcomes in HCC management.

It is essential to acknowledge the limitations of this study. The retrospective nature of our analyses based on multiple available datasets warrants further validation in cohorts with large sample to ensure robustness and generalizability. Furthermore, while our multi-omic analyses offer insights into the role of HMGB2 within the HCC and TME, further investigations are needed to elucidate its precise functional mechanisms. Despite these limitations, our study represents a significant contribution to understanding the complex interplay between HMGB2, HCC progression, and the tumor immune microenvironment. Leveraging multi-omics methods has allowed us to uncover novel insights into HCC pathogenesis and identify potential therapeutic targets, thereby benefiting patient outcomes and advancing clinical translational medicine.

## 5 Conclusions

In conclusion, our study highlights the pivotal role of HMGB2 in driving HCC aggressiveness and immunosuppression, offering new avenues for therapeutic intervention and patient stratification. By elucidating the intricate interplay between HMGB2 and the tumor microenvironment, we provide a foundation for the development of precision medicine approaches tailored to the unique molecular characteristics of individual tumors. Ultimately, the integration of HMGB2-targeted therapies into clinical practice holds promise for improving outcomes in patients with HCC.

## Data Availability

The datasets analyzed in this study are available from publicly accessible repositories, including The Cancer Genome Atlas (TCGA) (https://portal.gdc.cancer.gov/projects/TCGA-LIHC), the ICGC-LIRI cohort from the International Cancer Genome Consortium (ICGC) (https://dcc.icgc.org/projects/LIRI-JP), and Gene Expression Omnibus (GEO) (GSE144269, GSE109211, GSE104580, GSE14520, GSE54236, and GSE224411).
